# Influenza vaccine effectiveness in preventing hospitalisation of individuals 60 years of age and over with laboratory-confirmed influenza, Valencia Region, Spain, influenza season 2016/17

**DOI:** 10.2807/1560-7917.ES.2018.23.8.17-00318

**Published:** 2018-02-22

**Authors:** Ainara Mira-Iglesias, F Xavier López-Labrador, Beatriz Guglieri-López, Miguel Tortajada-Girbés, Víctor Baselga-Moreno, Laura Cano, Juan Mollar-Maseres, Mario Carballido-Fernández, Germán Schwarz-Chavarri, Javier Díez-Domingo, Joan Puig-Barberà, Valencia Hospital Network for the Study of Influenza and Respiratory Viruses Disease

**Affiliations:** 1 Fundación para el Fomento de la Investigación Sanitaria y Biomédica de la Comunitat Valenciana (FISABIO-Public Health), Valencia, Spain; 2 Consorcio de Investigación Biomédica de Epidemiología y Salud Pública (CIBERESP), Instituto de Salud Carlos III, Madrid, Spain; 3 Hospital Doctor Peset, Valencia, Spain; 4 Hospital Universitario y Politécnico La Fe, Valencia, Spain; 5 Hospital General Universitario de Castellón, Castellón, Spain; 6 Universidad CEU Cardenal Herrera, Castellón, Spain; 7 Hospital General de Alicante, Alicante, Spain; 8 Centro de Salud Pública de Castellón, Castellón, Spain

**Keywords:** influenza virus, surveillance, vaccine, epidemiology, hospitalisations, vaccine effectiveness

## Abstract

Seasonal influenza vaccination is widely recommended for people with risk factors, especially for people who are elderly. However, influenza vaccine effectiveness (IVE) varies year after year because of the variable antigenic composition of the circulating viruses and the vaccine composition. **Methods:** We summarise the results of IVE and the impact of previous vaccination among subjects 60 years of age and over in a multicentre prospective study in the Valencia Hospital Surveillance Network for the Study of Influenza and Respiratory Viruses Disease (VAHNSI) in Spain. We applied the test-negative design taking laboratory-confirmed influenza as outcome and vaccination status as exposure. Information about potential confounders was obtained from clinical registries or directly from patients. **Results:** Adjusted IVE was 19% (95% confidence interval (CI): −15 to 43). For patients vaccinated in the current season but not in the two previous seasons, effectiveness was 49% (95% CI: −20 to 78) and for patients vaccinated in the current and any of two previous seasons, effectiveness was 29% (95% CI: −3 to 52). For those patients not vaccinated in the current season but vaccinated in any of the two previous seasons, effectiveness was 53% (95% CI: 8 to 76). **Conclusions:** Our data show a low vaccine effectiveness for the 2016/17 influenza season.

## Introduction

Vaccination is the primary recommendation for preventing seasonal influenza worldwide [1,2]. It is highly recommended in people who are elderly as they represent around 90% of all influenza-related deaths [3,4]. The World Health Organization (WHO) and the European Council encourage vaccination among risk groups to reach a coverage of 75% in people who are elderly and those with underlying chronic conditions [5].

The continuous evolution of influenza viruses means that health authorities have to reformulate the vaccine composition each year, trying to predict which strains will circulate in the coming influenza season. In order to contribute to improvement and better understanding of influenza vaccines, since 2009, an active annual surveillance scheme in the Valencia Region in Spain has been monitoring influenza vaccine effectiveness (IVE) in preventing hospital admissions with laboratory-confirmed influenza [6-8].

In the 2016/17 influenza season, a trivalent vaccine containing the recommended A/Hong Kong/4801/2014(H3N2)-like virus [9] was offered free of charge to all Valencia Region inhabitants 60 years of age over. However, the 2016/17 season in the northern hemisphere was characterised by the circulation of A(H3N2) viruses belonging to a new emerging genetic subclade 3C.2a1, A/Bolzano/7/2016-like [10]. The new subclade is characterised by the N171K amino acid mutation as compared with the A/Hong Kong/4801/2014(H3N2)-like virus strain included in the northern hemisphere vaccine. In addition, significant amino acid heterogeneity in antigenic sites has been detected within this emerging subclade 3C.2a1 [11].

The interference between previous and current vaccination has been increasingly discussed. Castilla et al. [12] reported that whereas one or two vaccinations in the four prior seasons maintained or increased the protection of the current season vaccination, three or more doses had a negative effect on the current season vaccine. Other publications have suggested no protection with repeated vaccination, but that the current vaccination protected individuals unvaccinated in the previous season [13]. Similar results were obtained for the 2014/15 season in a school-based study in China [14] where children who had not received prior vaccination with a homologous A(H3N2) component were more protected than children receiving repeated vaccination. Other studies have explored the impact of repeated vaccination on IVE against A(H3N2) influenza. Two of them concluded that current season’s vaccination conferred either protection against medically attended influenza [15] or laboratory-confirmed hospitalised A(H3N2) influenza in people who are elderly [16], regardless of patients’ recent vaccination history. In our study, we report IVE estimates in preventing hospital admissions with laboratory-confirmed influenza during the 2016/17 influenza season in the Valencia Region in Spain. The impact of prior vaccination is also evaluated considering the two previous influenza seasons.

## Methods

### Study design

The study was carried out in four hospitals providing healthcare to 22% of the 4,860,874 inhabitants of Valencia Region in the east of Spain. The participating hospitals were: Hospital General de Castellón (Castellón, Spain), Hospital La Fe (Valencia, Spain), Hospital Doctor Peset (Valencia, Spain) and Hospital General de Alicante (Alicante, Spain).

Study procedures have been previously published [7]. In short, study staff screened consecutive hospital admissions through the emergency department. Patients were included in the study after written informed consent if they were a resident in the hospital catchment area, non-institutionalised, without a previous hospital discharge in the last 30 days and reported symptoms of influenza-like illness (ILI), defined as reported fever or feverishness, malaise, myalgia or headache and shortness of breath, sore throat or cough, within 7 days of admission. Subjects were considered immunised if they had received the current season’s influenza vaccine at least 14 days before symptoms onset.

The Ethics Research Committee of the Dirección General de Salud Pública-Centro Superior de Investigación en Salud Pública (DGSP-CSISP) approved the protocol of the study. All patients signed informed consent before the inclusion in the study.

### Vaccine information system

Information on the vaccine administered to all patients included in the study, in addition to the date of vaccination, was obtained from the Valencia Region Vaccine Information System (VRVIS). VRVIS is a population-based register that systematically records vaccine doses given at public and private vaccination points, including primary care centres, hospitals, residential facilities in the public sector and any private sector facility that applies for access. The sensitivity and specificity of VRVIS was estimated to be 90% and 99%, respectively [7,17]. All registered residents of Valencia Region have a unique identification number that is linked to the VRVIS, inpatient and outpatient clinical records, and sociodemographic information. Vaccine information was ascertained by recall in those patients whose vaccine administration was not registered in the system.

### Laboratory procedures

Nasopharyngeal and pharyngeal swabs were collected from patients meeting the inclusion criteria and tested by real-time reverse transcription-PCR (RT-PCR) for influenza. The specimens were tested in a centralised virology laboratory at Fundación para el Fomento de la Investigación Sanitaria y Biomédica de la Comunitat Valenciana (FISABIO-Public Health) following World Health Organization (WHO) protocols [18]. In brief, one-third of the viral transport media volume in the combined swab sample tubes was extracted for total nucleic acids using an automated silica-based method (NucliSENS easyMAG, BioMérieux, Marcy-l’Etoile, France). A multiplex real-time RT-PCR screening assay was used to detect influenza A and B viruses using different primers and probes for the matrix protein [19,20] and the qScript XLT One-Step RT-qPCR ToughMix (Quantabio, Beverly, Massachusetts, United States (US)) in a LightCycler 480II apparatus (Roche Molecular Diagnostics, Sant Cugat, Spain).

For haemagglutinin (HA) sequencing, all isolates from hospitalised cases with sufficient viral load (cycle threshold (Ct) ≤ 26) were systematically selected and a specific RT-PCR amplification protocol was applied using gene-specific primers for the corresponding virus subtype [21]. The amplified fragments were sequenced by the Sanger method with the BigDye Direct Cycle Sequencing Kit in an ABI 3730xl DNA Sequencer (Applied Biosystems, Life Technologies, Foster City, California, US) at the Genomics Core of the Servei Central de Suport a la Investigació Experimental (SCSIE), University of Valencia, Spain.

### Molecular phylogenetic analysis of influenza A(H3N2) viruses

Because we detected no influenza B viruses and only one A(H1N1)pdm09 virus during the 2016/17 season, the phylogenetic characterisation was performed in A(H3N2) viruses only. A dataset with HA sequences was constructed including the obtained influenza virus HA sequences together with representative and reference HA sequences (Supplement 1) obtained from the Global Initiative on Sharing All Influenza Data (GISAID) database (www.gisaid.org). These representative sequences were chosen for specific amino acid signatures in the HA gene defining known phylogenetic clades. The dataset alignment was generated with the ClustalW algorithm integrated in the BioEdit software version 7.2.5 (http://www.mbio.ncsu.edu/bioedit/bioedit.html). Phylogenetic trees were inferred using maximum likelihood methods and the best-fitting nt substitution model (general time-reversible with gamma distribution among sites) with the online PhyML 3.0 platform (http://www.atgc-montpellier.fr/phyml). Branch reliability was evaluated by approximate likelihood-ratio tests [22]. The tree was rooted on the A/Perth/16/2009(H3N2), clade 3C virus.

### Statistical analysis

Characteristics of influenza-positive hospital admissions (cases) and influenza-negative hospital admissions (controls) were compared performing a chi-squared test. We used the same test when comparing vaccinated and unvaccinated individuals. All probabilities were two-tailed and p values under 0.05 were considered statistically significant.

The test-negative design was used to estimate the ratio of the odds of vaccination among individuals testing positive for influenza to the odds of vaccination among individuals testing negative. This approach has been described elsewhere [23,24] since it is less susceptible to bias due to misclassification of infection and to confounding by healthcare-seeking behaviour in comparison with case–control studies. Cases were patients testing positive for influenza and controls were patients who tested negative. The adjusted odds ratio (aOR) was estimated using a mixed effects logistic regression model including age, sex, number of underlying chronic conditions, previous hospital admissions in the last 12 months, general practitioner (GP) consultations in the last 3 months, smoking habits, socioeconomic class according to occupation [25], days from onset of symptoms to swabbing and hospital as fixed effects, and epidemiological week at admission as random effect. Including epidemiological week as random effect allowed us to control for considerable extra variation that could be present across the different study weeks (number of working days, holidays and risk of infection). IVE was calculated as (1 − aOR) × 100. The same estimates were performed according to current and prior two seasons influenza vaccination taking as reference category no vaccination in any of the seasons.

All statistical analyses were carried out in Stata version 14 (StataCorp, College Station, Texas, US).

## Results

### Study subjects

**Figure 1:**
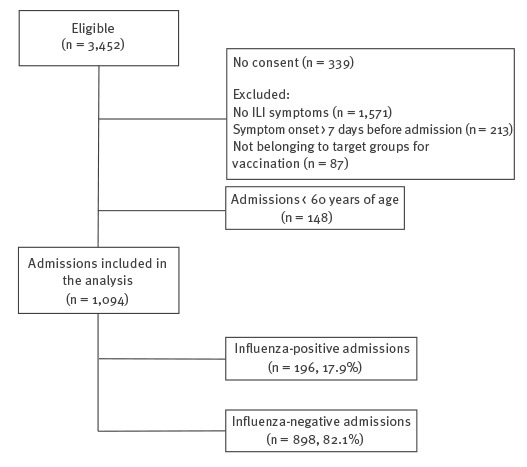
Flowchart of hospital admissions 60 years of age and over included in the influenza vaccine effectiveness analysis, Valencia Hospital Network for the Study of Influenza (VAHNSI), Valencia Region, Spain, influenza season 2016/17 (n = 1,094)

The analysis was restricted to individuals 60 years of age and over because of small numbers in the other age groups. After applying exclusion criteria, 1,094 hospital admissions were included in the analysis (Figure 1). Vaccination was ascertained by recall (13% of included patients) and by consulting the VRVIS (87% of included patients).

### Study period

**Figure 2:**
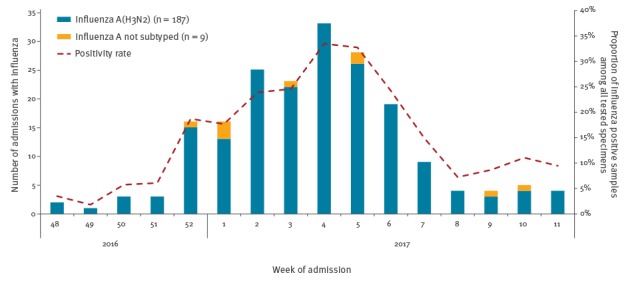
Distribution of influenza cases aged 60 years and over by influenza type/subtype and positivity rate by epidemiological week, Valencia Hospital Network for the Study of Influenza (VAHNSI), Valencia Region, Spain, influenza season 2016/17 (n = 196)

The beginning of the influenza season was defined as the first of two consecutive weeks with two or more influenza cases, and the end as the previous week to the first of two consecutive weeks with no influenza cases (Figure 2). This period was from week 48 of 2016 (starting 28 November) to week 11 (starting 13 March) of 2017. Our first influenza-positive patient was admitted on 29 November 2016 and our last influenza-positive patient was admitted 17 March 2017.

### Influenza cases vs controls

A total of 196 hospital admissions (17.9%) were positive for influenza A: 187 (95.4%) were influenza A(H3N2) and 9 (4.6%) remained unsubtyped because of low viral loads (Table 1).

**Table 1:** Characteristics of hospital admissions aged 60 years and over included in the influenza vaccine effectiveness analysis, Valencia Hospital Network for the Study of Influenza (VAHNSI), Valencia Region, Spain, influenza season 2016/17 (n = 1,094)

Characteristics	Influenza-positive admissions (cases)		Influenza-negative admissions (controls)		p value^a^	Vaccinated 2016/17			
	n	%	n	%		n	Total	%	p value^b^
Overall	196	17.9	898	82.1	–	648	1,094	59.2	–
**Age**									
60–69 years	21	10.7	180	20.0	**0.004**	94	201	46.8	**<0.001**
70–79 years	62	31.6	304	33.9		212	366	57.9	
80–89 years	85	43.4	330	36.7		267	415	64.3	
≥ 90 years	28	14.3	84	9.4		75	112	67.0	
**Sex**									
Male	93	47.4	475	52.9	0.167	341	568	60.0	0.574
Female	103	52.6	423	47.1		307	526	58.4	
**Number of underlying chronic conditions**									
None	26	13.3	110	12.2	0.628	62	136	45.6	**<0.001**
One	59	30.1	246	27.4		170	305	55.7	
Two or more	111	56.6	542	60.4		416	653	63.7	
**Hospital admission in the last 12 months**									
Yes	53	27.0	345	38.4	**0.003**	257	398	64.6	**0.007**
No	143	73.0	553	61.6		391	696	56.2	
**Number of general practitioner consultations in the last 3 months**									
None	28	14.3	103	11.5	0.090	65	131	49.6	**<0.001**
One	49	25.0	177	19.7		108	226	47.8	
Two or more	119	60.7	618	68.8		475	737	64.5	
**Smoking habits**									
Never	128	65.3	472	52.6	**0.002**	359	600	59.8	**<0.001**
Ex-smoker	54	27.6	303	33.7		228	357	63.9	
Current smoker	14	7.1	123	13.7		61	137	44.5	
**Time from symptoms onset to swabbing**									
0–2 days	26	13.3	144	16.0	0.108	109	170	64.1	0.287
3–4 days	97	49.5	359	40.0		273	456	59.9	
5–7 days	56	28.6	297	33.1		205	353	58.1	
> 7 days	17	8.7	98	10.9		61	115	53.0	
**Influenza test results**									
Negative	–	–	898	100.0	–	537	898	59.8	0.414
A(H3N2)	187	95.41	–	–		107	187	57.22	0.538
A not subtyped	9	4.59	–	–		4	9	44.44	0.365
**Vaccinated 2016/17**	111	56.6	537	59.8	0.414	–	–	–	–
**Vaccinated 2015/16**	101	51.5	541	60.2	**0.025**	551	642	85.8	**< 0.001**
**Vaccinated 2014/15**	104	53.1	519	57.8	0.225	525	623	84.3	**< 0.001**
**Registration in the Valencia Region Vaccine Information System**	161	82.14	786	87.5	**0.045**	643	947	67.9	**< 0.001**

^a^ p value comparing influenza-positive admissions and influenza-negative admissions.

^b^ p value comparing vaccinated with unvaccinated admissions.

Individuals testing positive for influenza were notably older than those testing negative. The percentage of admissions in hospital during the previous year among influenza-positive admissions was lower than the percentage among influenza-negative admissions. In terms of smoking habits, influenza-negative admissions were more likely to be current or ex-smokers than those who were influenza-positive. The proportion of vaccinated individuals in the 2015/16 season was significantly higher in those testing negative for influenza (Table 1).

### Vaccinated vs unvaccinated hospital admissions

A total of 648 (59.2%) hospital admissions were vaccinated for the 2016/17 influenza season. As expected, vaccination coverage increased significantly with age, but also with the number of underlying conditions. Vaccinated admissions were more likely admitted during the last year and to visit the GP in two or more occasions in the last 3 months. Admissions who never smoked or ex-smokers were more vaccinated than current smokers. Most of the admissions vaccinated in the current season were also vaccinated in the 2015/16 and in the 2014/15 influenza seasons (Table 1).

### Molecular analysis

**Figure 3:**
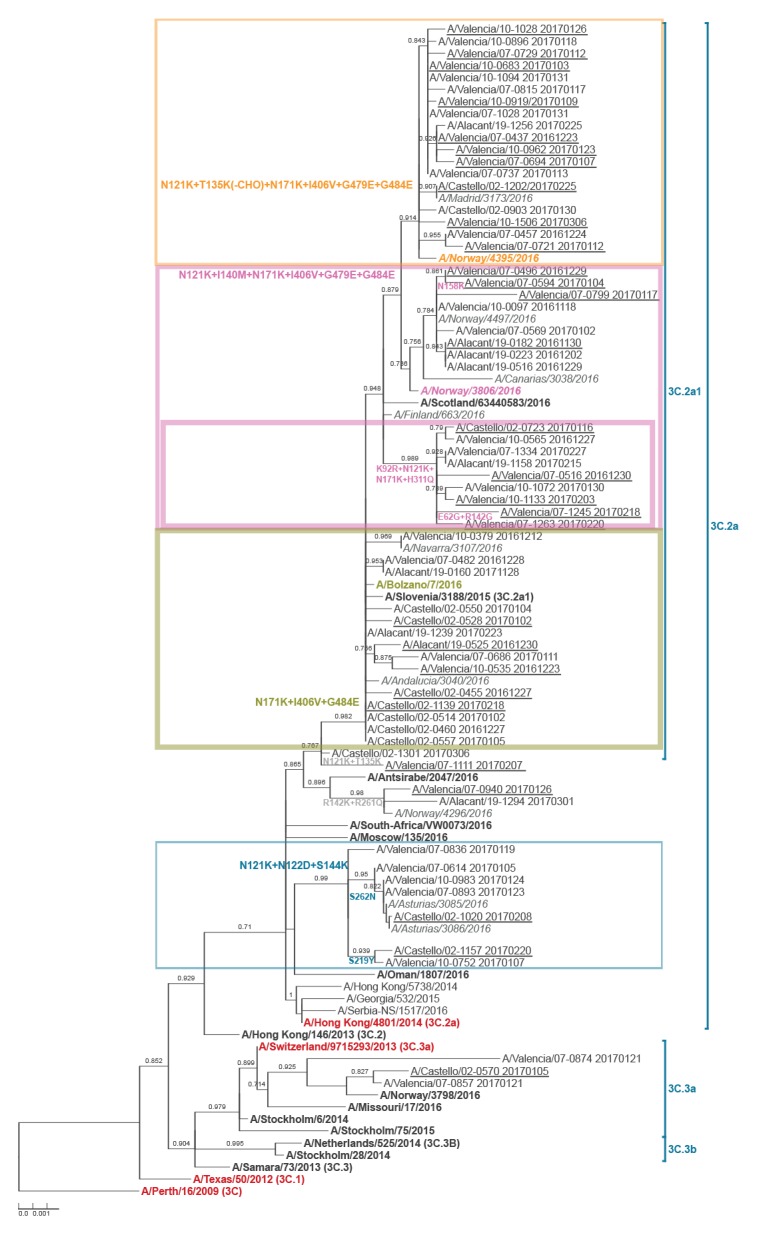
Phylogenetic tree of influenza A(H3N2) isolates, Valencia Hospital Network for the Study of Influenza (VAHNSI), Valencia Region, Spain, influenza season 2016/17

The genetic characterisation of the 63 sequenced influenza A(H3N2) samples indicated circulation of few clade 3C.3a, A/Switzerland/9715293/2013-like, viruses (n = 3), with the majority of isolates (n = 60), corresponding to the same clade (3C.2a) as the A/Hong Kong/4801/2014 vaccine strain for the 2016/17 season (Figure 3).

Phylogenetic tree obtained by using maximum likelihood methods (general time-reversible substitution model with gamma distribution among sites) with PhyML 3.0. Branch reliability was evaluated by approximate likelihood-ratio tests and the tree was rooted on the A/Perth/16/2009(H3N2), clade 3C virus. Reference sequences from the GISAID database. Red indicates vaccine strains, bold black indicates clade reference strains, italics represent European A(H3N2) isolates circulating during the 2016/17 influenza season, not related to this study. Local isolates from vaccinated individuals are underlined. The four amino acid variation patterns within subclade 3C.2a1 viruses are highlighted by different boxes. A new 3C.2a local clade is highlighted by a blue coloured box.

The authors gratefully acknowledge the originating and submitting laboratories who contributed sequences used in the phylogenetic analysis to the Global Initiative on Sharing All Influenza Data (GISAID, www.gisaid.org).

However, 82% (n = 49) of these clade 3C.2a viruses belong to subclade 3C.2a1, all characterised by the N171K mutation as compared with the A/Hong Kong/4801/2014 vaccine strain for the 2016/17 season (Figure 3). In addition, within this subclade 3C.2a1 we observed significant amino acid heterogeneity in antigenic sites, with up to four groups each characterised by a particular subset of mutations: (i) A/Bolzano/7/2006-like group (n = 14): N171K(HA1), I406V + G484E(HA2); (ii) a local cluster (n = 9) with additional mutations K92R(site E) + N121K(site D) + H311Q(site C) (HA1); (iii) A/Norway/3806/2016-like group (n = 8): N121K(site D) + I140M + N171K(HA1), I406V + G479E + G484E(HA2); and (iv) A/Norway/4395/2016-like group (n = 18): 121K(site D) + T135K(site A) + N171K(HA1), I406V + G479E + G484E(HA2); the latter with a potential loss of glycosylation at position 135. Finally, the remaining eleven clade 3C.2a isolates were also genetically heterogeneous. Four were related to A/Antsirabe/2047/2016, with either mutations N121K(site D) + T135K(site A) or R142K(site A) + R261Q(site E), and the rest belonged to a new cluster characterised by the N121K(site D) + N122D(site A) + S144K(site A) and either S219Y or S262N(site E) (all HA1) mutations (Figure 3). The distribution of viral isolates from vaccinated individuals along the phylogenetic tree did not reveal any significant aggregation of cases with any particular virus genetic group.

### Influenza vaccine effectiveness and impact of prior vaccination

Overall IVE in preventing hospital admissions with influenza was 18.7% (95% confidence interval (CI): −15.1 to 42.5) regardless of previous vaccination status. By age group, IVE was 37.0% (95% CI: −91.4 to 79.3) in 60–69 years of age admissions, −33.8% (95% CI: −158.0 to 30.6) in those 70–79 years of age, 27.3% (95% CI: −29.1 to 59.1) in those 80–89 years of age and 61.0% (95% CI: −31.0 to 88.4) in those 90 years of age and over (data not shown).

To explore the impact of prior vaccination, we took individuals unvaccinated in the 2016/17 influenza season and two previous seasons as reference. For patients vaccinated in the current season but not in the two previous seasons, IVE was 48.6% (95% CI:−20.0 to 78.0); for patients vaccinated in the current season and in either or in both of the two previous seasons, IVE was 29.4% (95% CI:−3.0 to 51.6); and for those not vaccinated in the current season but in either or both of the two previous seasons, IVE was 53.5% (95% CI: 8.2 to 76.4) (Table 2).

**Table 2:** Adjusted influenza vaccine effectiveness against hospital admission of individuals 60 years old or of age and over with laboratory-confirmed influenza, Valencia Hospital Network for the Study of Influenza (VAHNSI), Valencia Region, Spain, influenza season 2016/17

Analyses	Cases (n = 196)		Controls (n = 898)		Adjusted IVE^a^	
	Vaccinated	%	Vaccinated	%	VE %	95% CI
Overall 2016/17^b^	111	56.6	537	59.8	18.7	−15.1 to 42.5
NV in the previous two seasons, V in the current season^c^	8	4.1	53	5.9	48.6	−20.0 to 78.0
V in either or both previous two seasons, V in the current season^c^	103	52.6	484	53.9	29.4	−3.0 to 51.6
V in either or both previous two seasons, NV in the current season^c^	13	6.6	109	12.1	53.5	8.2 to 76.4

V: vaccinated, NV: not vaccinated, IVE: influenza vaccine effectiveness.

^a^ Adjusted by age, sex, number of underlying chronic conditions, previous hospital admissions in the last 12 months, general practitioner consultations in the last 3 months, smoking habits, socioeconomic class, days from onset of symptoms to swabbing and hospital as fixed effects, and epidemiological week at admission included as a random effect.

^b^ Comparing vaccinated and unvaccinated admissions in the current influenza season.

^c^ Admissions unvaccinated in the current and previous two seasons taken as reference.

## Discussion

The 2016/17 influenza season in the Valencia Region of Spain was dominated by influenza A viruses with almost all subtyped as A(H3N2). The positivity peak was earlier than those in recent years, and was higher than those observed in the 2015/16 and 2013/14 influenza seasons but similar to that observed in the 2014/15 season, which was also A(H3N2)-dominant [11]. Most of the genetically characterised viral isolates in Valencia Region belonged to a new emerging subclade, 3C.2a1, evolving from the A/Hong Kong/4801/2014(H3N2) northern hemisphere 2016/17 influenza vaccine strain. Previous preliminary studies have also found these variant viruses in their analyses [26-28]. This emerging subclade has been reported as antigenically like the vaccine virus [10,11,27] and preliminary IVE estimates have been moderate to low [12,26-28]. However, 3C.2a1 viruses are difficult to characterise antigenically as, due to accumulated mutations, there is a lack of appropriately matched reference antisera and agglutination capacity in haemagglutination assays is reduced [11].

Our results corroborated that this new emerging 3C.2a1 subclade was genetically heterogeneous. We distinguished at least three genetic subgroups with different amino acid mutation patterns in antigenic sites A, C and D as compared with the A/Hong Kong/4801/2014(H3N2) vaccine virus, one of them with the potential loss of a glycosylation site (T135K), which has also been found in Canada [26]. In addition, we identified a novel 3C.2a local clade in Spain with three to four mutations in antigenic sites A, D and E (characterised by N121K + N122D + S144K) as compared with the vaccine strain, with potentially modified antigenicity. Interestingly, similar new 3C.2a viruses have been characterised recently in the United Kingdom in the context of an outbreak [29].

Collectively, our results and available data published elsewhere suggest that clade 3C.2a genetic diversification is ongoing, with rapid dynamics of different subclades. Although no significant changes in serological data have been detected yet, the diverse mutation patterns of new subclades suggest distinct mutant viruses can become predominant during the next 2017/18 season, whereas the recommended vaccine strain is still the same A/Hong Kong/4801/2014(H3N2)-like virus. Whether the new predominant A(H3N2) viruses in the next 2017/18 season will be associated with changes in antigenicity and vaccine effectiveness deserves close monitoring, as the potential implications for Public Health may be important. The sensitivity to oseltamivir of the majority of the 2016/17 A(H3N2) viruses may warrant antiviral management when necessary [11].

Our IVE analysis was restricted to admissions 60 years of age and over because of small numbers in the other age groups. Other studies reported that confirmed cases of influenza A were predominant in adults aged over 65 years of age with a substantial increase in mortality among individuals in this age group [26]. We observed a significant residual protection of previous vaccination and a lower effect in those vaccinated only in the current season or in the current and any of the two previous seasons. This pattern of protection was consistent with preliminary estimates in Europe [12,28].

Our results showed that IVE estimates were lower in the primary analysis, when no current vaccination rather than no current/no prior vaccination was used as reference group. This may be suggestive of residual protection (IVE for prior vaccination only). Interestingly, this residual protection may include vaccination with the previous A(H3N2) vaccine strain A/Switzerland/9715293/2013 used in 2015/16 season, perhaps because of some cross-reactivity. Because of the residual protection observed, our findings do not support that a possible antigenic mismatch among the A/Hong Kong/4801/2014 vaccine strain and the 2016/17 circulating strains influenced IVE according to the antigen distance hypothesis [30]. However, we and others [26-28] observed discrepancies between antigenic and vaccine effectiveness data that reinforce the consideration of genetic sequence analyses of influenza viruses [31].

Our study had several limitations. The results regarding the impact of repeated vaccination in the presence of genetic variation [32] were reassuring but should be interpreted with caution. The absence of statistical significance is expected in studies with low vaccine coverage, moderate to low effectiveness or a limited sample size resulting in wide confidence intervals [33]. The observational nature of our study meant that there was some heterogeneity in inpatient settings due to case ascertainment and exposure. However, we restricted the analysis to periods with influenza circulation and only included patients with ILI and an onset of symptoms within 7 days of admission to account for this heterogeneity. In addition, vaccination status was mostly ascertained with the information obtained from the VRVIS, and influenza positivity with a sensitive RT-PCR assay. Finally, we only included those patients with available swabbing performed within 48 hours of admission into hospital to avoid classification bias.

## Conclusion

Although circulating influenza viruses were from the same genetic clade as the vaccine virus (A/Hong Kong/4801/2014), they showed a remarkable genetic heterogeneity with changes in antigenic sites. New 3C.2a subclades showed rapid and ongoing diversification, with important implications for the next 2017/18 influenza season because potential changes in antigenicity together with use of the same 2016/17 A/Hong Kong/4801/2014(H3N2)-like vaccine strain. Very early characterisation of A(H3N2) viruses will be necessary for changes in vaccine effectiveness during the 2017/18 influenza season [11], as shown by recent data from Canada [34].

The estimated IVE was low, although not significant, when comparing vaccinated and unvaccinated individuals in the current season regardless recent vaccination history. When considering prior vaccination, patients vaccinated in any of the two previous seasons but not in the current one were more protected than those individuals vaccinated in the current but not in the previous two seasons suggesting a residual protection obtained from previous vaccinations.

This work adds valuable information to the study of IVE and the controversial issue about the impact of repeated influenza vaccination. Our data may be especially relevant considering that vaccination against laboratory-confirmed influenza is being highly recommended in an increasingly number of countries worldwide.
